# Association of insulin resistance indices with kidney stones and their recurrence in a non-diabetic population: an analysis based on NHANES data from 2007–2018

**DOI:** 10.1080/0886022X.2025.2490203

**Published:** 2025-04-24

**Authors:** Yu-Xuan Yang, Jia-Cheng Xiang, Gui-Chen Ye, Kuang-Di Luo, Shao-Gang Wang, Qi-Dong Xia

**Affiliations:** Department and Institute of Urology, Tongji Hospital, Tongji Medical College, Huazhong University of Science and Technology, Wuhan, China

**Keywords:** Insulin resistance indices, NHANES, insulin resistance, kidney stones

## Abstract

**Objective:**

To systematically evaluate the association between insulin resistance indices and the risk of kidney stones and their recurrence in U.S. non-diabetic individuals, while identifying predictive indicators.

**Materials and Methods:**

This cross-sectional study analyzed data from the 2007–2018 NHANES. Five IR indices were calculated. Weighted logistic regression, restricted cubic spline, and mediation analyses were used to assess the independent associations between these indices and the risk of kidney stones and recurrence in non-diabetic individuals.

**Result:**

This study of 9,605 non-diabetic participants showed an overall kidney stones incidence of 8.63% and a recurrence rate of 2.70%. Weighted logistic regression and RCS analyses revealed significant positive associations between METs-IR, HOMA-IR, TyG-BMI, and the risk of kidney stones and their recurrence. Every unit increase in METs-IR was linked to a 2% rise in the incidence of kidney stones (95% CI: 1.014–1.027, *p* < 0.001) and a 3.3% rise in recurrence (95% CI: 1.018–1.048, *p* < 0.001); each unit increase in HOMA-IR raised incidence by 5% (95% CI: 1.025–1.078, *p* < 0.001) and recurrence by 7.9% (95% CI: 1.041–1.118, *p* < 0.001). Adjusting for confounders shifted these relationships from nonlinear to linear (*p* > 0.05). METs-IR demonstrated the strongest diagnostic accuracy for predicting recurrence, with uric acid and vitamin D mediating associations between IR indices and the risk of kidney stones and their recurrence in non-diabetic individuals.

**Conclusion:**

This study found that elevated IR indices (METs-IR, HOMA-IR, TyG-BMI) significantly increased kidney stone risk in a non-diabetic population. Serum uric acid and vitamin D mediated this association, with METs-IR best predicting kidney stones incidence and recurrence.

## 1. Introduction

Kidney stones represent one of the most prevalent afflictions of the urinary system worldwide, affecting millions of individuals and approximately 10% of the U.S. population [1,2]. This complex disease not only reduces quality of life but also imposes a substantial healthcare burden [3]. The formation of kidney stones is a complex process that involves multiple factors, including metabolic abnormalities, genetics, lifestyle, dietary habits, and environmental factors [4]. In recent years, growing evidence has highlighted a strong association between metabolic disorders, particularly insulin resistance (IR), and the development of kidney stones [1,5]. As a key feature of metabolic syndrome, IR reflects the body’s diminished sensitivity to insulin, resulting in disrupted glucose metabolism [6]. Moreover, IR affects urinary metabolism, such as reducing renal tubular ammonia production and altering urinary pH [7,8]. These metabolic imbalances represent a significant contributing factor to kidney stones formation.

The hyper-insulinemic euglycemic clamp is currently recognized internationally as the definitive method for evaluating insulin resistance, but owing to its high price and cumbersome procedures, it is currently only used in scientific research and cannot be used on a large scale in clinical practice [9]. At this juncture, the most commonly utilized clinical indices for the evaluation of insulin resistance are the homeostasis model assessment (HOMA-IR) [10], the metabolic index of insulin resistance score (METs-IR) [11], the triglyceride glucose index (TyG) [12], and the triacylglycerol/high-density lipoprotein cholesterol (TG/HDL-C) ratio [13], which have been significantly correlated with the results of the gold standard. Among them, TyG index is regarded as a convenient and fairly straightforward indicator of IR due to its low computational cost and easy availability [14]. In contrast, HOMA-IR has limited its use in clinical practice due to its dependence on fasting insulin measurements [15]. Recent studies have shown that intergrading the TyG index with body mass index (TyG-BMI) enhances its accuracy in assessing insulin resistance (IR) significantly [16].

However, most studies on the connection between IR index and kidney stone risk have focused on diabetic populations and have been limited to assessing the impact of individual indices on populations, while relatively few large-scale comprehensive studies have been conducted in non-diabetic populations. In the non-diabetic population, the insulin resistance index may represent an under-appreciated risk factor for kidney stones. In this study, five insulin resistance indices, including TyG index, HOMA-IR, TG/HDL-C, METs-IR and TyG-BMI, will be utilized to explore the association of these indices with kidney stones and their recurrence in a non-diabetic population in America. This study is designed to address the existing knowledge gap and to gain deeper insights into their predictive effects. To ensure the broad applicability of our findings, we chose to analyze data from the nationally representative NHANES dataset, which provides generalizable conclusions for the U.S. population. NHANES includes rich demographic, metabolic, and lifestyle data, making it ideal for studying the complex relationship between IR and kidney stones. By leveraging this dataset, our study offers valuable insights into the predictive effects of IR indices on kidney stone risk and lays a scientific foundation for personalized prevention strategies.

## 2. Material and methods

### 2.1. Population sample

**Figure 1.**
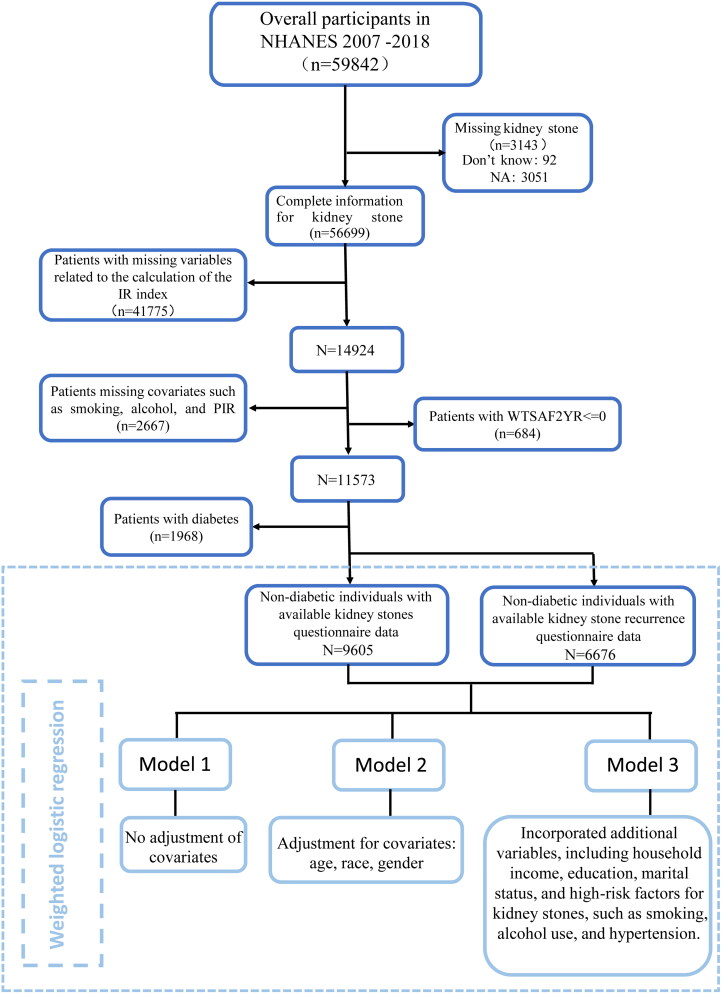
Flow diagram of the inclusion and exclusion criteria from national health and nutrition examination survey (NHANES) 2007–2018.

This study utilized a cross-sectional design based on the NHANES dataset, which is accessible for retrieval from the official NHANES website (https://www.cdc.gov/nchs/nhanes/Default.aspx). All components of the study were granted approval by the Review Board at the NCHS, and all participants voluntarily gave informed consent for the study. A total of 59,842 participants from 2007–2018 were recruited for this study, and the exclusion criteria were (1) missing data on kidney stones; (2) missing indicators related to the calculation of the Insulin Resistance Index: fasting blood glucose, BMI, and glycosylated hemoglobin; (3) missing covariates; (4) weight in fasting subsample (WTSAF2YR) ≤ 0. From these, 11,573 participants were screened, and since the population discussed in this study was a non-diabetic population, we defined diabetes according to American Diabetes Association (ADA) criteria, with a diagnosis made by meeting one of the following criteria: HbA 1 *C* ≥ 6.5%; FPG ≥ 7.0 mmol/L (126 mg/dL); self-reported diabetes and the use of oral hypoglycemic medications or insulin, to screen out the non-diabetic population [17]. Ultimately, 9605 non-diabetic participants met the criteria for inclusion in the full case analysis. The detailed screening process is described in Figure 1.

### 2.2. Outcome

A history of kidney stones was defined based on participants’ responses to the question, ‘Have you ever had kidney stones?’ question (NHANES 2007–2018). Adult participants who answered ‘yes’ were considered to have a history of kidney stones. Because the NHANES database only provides data on kidney stone recurrence from 2007–2014, only kidney stone recurrence during that time period was analyzed in this study. The definition of kidney stone recurrence was based on the question ‘How many times have you had kidney stones?’ question (NHANES 2007–2014). Any patient who answered more than twice was considered to have had a kidney stone recurrence.

### 2.3. Exposure

This study incorporated five indices to assess insulin resistance, including TyG index, TyG-BMI, HOMA-IR, TG/HDL-C and METs-IR. These indices were selected for their established clinical relevance, simplicity of calculation, and accessibility in large-scale population studies. Among them, HOMA-IR is widely regarded as a traditional IR marker, calculated as fasting glucose (mmol/L) × fasting insulin (mIU/L)/22.5 [10]; However, its reliance on fasting insulin measurements limits its routine use in clinical practice due to the complexity and cost of insulin assays. TyG index and its expanded form TyG-BMI, defined as Ln [fasting glucose (mg/dL) × Triglycerides (mg/dL)/2] and Ln [fasting glucose × Triglycerides/2] × BMI, respectively [18,19], offer cost-effective and straightforward proxies for IR, derived from routinely available metabolic markers. TyG-BMI, in particular, has been shown to enhance accuracy by incorporating body composition into the assessment. METs-IR, calculated as Ln[2 × fasting glucose (mg/dL) + fasting triglycerides (mg/dL)] × BMI/(Ln(HDL-C))(mg/dl) [11], provides a comprehensive measure by integrating multiple metabolic parameters, making it valuable for predicting metabolic and cardiovascular disorders. Similarly, TG/HDL-C, derived as Triglycerides (mg/dL)/HDL-C (mg/dL) [20], serves as a simple surrogate for atherogenic dyslipidemia, with strong correlations to IR and related complications. The five indices chosen in this study are well-validated, highly correlated with the gold standard (hyper-insulinemic euglycemic clamp), and readily applicable to NHANES data, enabling us to include a broad population, which enhances the statistical power and representativeness of our study. By selecting these indices, this study ensures a balance between reliability, accessibility, and clinical relevance, enabling a comprehensive evaluation of IR and its association with kidney stones formation and recurrence.

### 2.4. Assessment of covariates

Based on previous literature, we collected several additional covariates from the NHANES database adjusted according to the four dimensions of demographics, physical examination, laboratory examination, and questionnaire as a way to minimize bias from previous studies [21,22]. The final covariates included in this study were age, gender, race, BMI, household income, educational status, hypertension status, drinking and smoking status. Age was divided into three categories: young (20–35 years), middle-aged (35–65 years), and old (>65 years); race was classified as non-Hispanic white, non-Hispanic black, Mexican American, other Hispanic, or other categories; household earning PIR was categorized as low-income (<1), middle-income (1–3), and high-income (>3); educational level was categorized as below high school, college or above and high school. In addition, BMI was categorized into 3 groups according to the standardized thresholds of the World Health Organization, with BMI <18.5 kg/m^2^ being underweight; BMI > =25 kg/m^2^ being overweight; and the rest being normal weight [23]. Smoking status was defined as having a history of smoking ‘more than 100 cigarettes in one’s lifetime’, while drinking status was defined as ‘consuming at least 12 alcoholic drinks in the past year’. Hypertension was identified by the presence of any of the following criteria: average diastolic blood pressure (DBP) ≥ 90 mmHg or average systolic blood pressure (SBP) ≥ 140 mmHg, use of prescription antihypertensive medications, or self-reported hypertension [24].

As vitamin D and uric acid have demonstrated a strong association with the risk of kidney stones [25,26], in order to explore whether these two items mediate the connection between IR and kidney stones as well as kidney stone recurrence, we decided to include them as covariates.

### 2.5. Statistical analysis

In this study, based on the principle of using the NHANES minimum subgroup weights [27], fasting subsample weights were selected to account for the NHANES complex survey design, as the dataset included fasting data. For kidney stone data from 2007 to 2018, twelve-year weights were constructed by dividing WTSAF2YR by 6. Since the kidney stone recurrence data only covered the period from 2007 to 2014, eight-year weights were constructed by dividing WTSAF2YR by 4. A table of baseline characteristics was constructed by stratifying demographic characteristics of the study participants and their corresponding laboratory parameters according to quartiles (Q1-Q4) using five proxy indicators of IR (TyG-BMI, HOMA-IR, METS-IR, TyG index and TG/HDL-C), as independent variables. Categorical variables were represented as weighted percentages, while continuous variables were expressed as weighted means with Standard Error (SE). For comparisons of continuous variables, a weighted t-test was used for two-group comparisons, while weighted linear regression models were applied for multiple-group comparisons. Overall p-values were obtained using the Wald test. For categorical variables, weighted chi-square tests were conducted to assess between-group differences, ensuring that all statistical analyses accounted for the complex survey design. Furthermore, the cohort was divided into renal stone and non-renal stone groups based on patient prognosis, as well as renal stone recurrence and no recurrence groups, and their baseline characteristics were compared using the same statistical methods. Three logistic regression models were employed to evaluate the association between IR indices and kidney stones as well as kidney stone recurrence. Model 1 did not include covariates; Model 2 included adjustments for basic participant characteristics, specifically age, gender, and ethnicity; Model 3 incorporated additional variables, such as household income, poverty-to-income ratio (PIR), education level, marital status, and high-risk factors for kidney stone formation, including smoking, alcohol consumption, hypertension and serum uric acid levels. To further explore the potential nonlinear relationships between IR indices and kidney stone outcomes, we employed restricted cubic spline (RCS) analysis. Unlike traditional methods that assume a simple linear relationship, RCS allows for a more nuanced evaluation. For example, this approach can identify inflection points where the risk of kidney stones changes more sharply or plateaus, offering deeper insights into how IR indices influence kidney stone formation and recurrence. We then conducted a mediation analysis using the ‘mediation’ package in R to assess the potential mediating role of uric acid and vitamin D levels in this association, with indirect effects estimated through 1,000 bootstrapping iterations. Additionally, we explored the predictive value of IR indices for kidney stones formation and recurrence using ROC curves. Finally, to assess the robustness of the correlation, subgroup analyses and interaction assessments were conducted across various subgroups. All statistical analyses were conducted utilizing R (4.3.3), and at a *p* < 0.05, statistical significance was established. This work has been reported in line with the STROCSS criteria [28].

## 3. Results

### 3.1. Baseline characteristics of participants

This study included 9,605 non-diabetic participants in total, and the participants were categorized into four groups based on two criteria: the presence or absence of kidney stones, and whether they had experienced recurrent kidney stones or not, and the detailed demographic characteristics and laboratory findings are shown in Table 1. The overall incidence of kidney stones was 8.63% (*n* = 829), with a recurrence rate of 2.70% (*n* = 180). Insulin resistance-related indices (TyG index, METs-IR, TG/HDL-C, HOMA-IR, and TyG-BMI index) were observed to be notably higher in the groups with kidney stones incidence and recurrence compared to the groups with no kidney stones and no recurrence (*p* < 0.001). This suggests a significant association between insulin resistance and the incidence and recurrence of kidney stones. Uric acid levels were significantly elevated in the kidney stones group (*p* = 0.0013), yet no significant correlation was observed with recurrence (*p* = 0.0675). Additionally, vitamin D levels did not exhibit notable differences between the groups. Furthermore, gender, age, race, weight status, and hypertension were identified as strongly linked to both the initial emergence and reappearance of kidney stones (*p* < 0.05). The group of individuals older than 65 years of age exhibited a higher proportion of kidney stones and recurrence (19.25%; 19.88%). The analysis revealed a notable link between marital status and the recurrence of kidney stones (*p* = 0.001), indicating a higher recurrence rate among unmarried individuals. No significant differences were observed in the distribution of education level, poverty-income ratio, smoking, and alcohol consumption between the kidney stone and recurrence groups (*p* > 0.05).

**Table 1.** Baseline characteristics of participants weighted for demographic as well as clinical characteristics based on the presence or absence of a diagnosis of kidney stones and the presence or absence of kidney stone recurrence.

Characteristic	Kidney stones					Kidney stone recurrence				
	Overall, N	Overall	Yes (*n* = 829)	No (*n* = 8776)	*P* Value	Overall, N	Overall	Yes (*n* = 180)	No (*n* = 6496)	*P* Value
TyG index	9605	8.54 (0.00910)	8.63 (0.0229)	8.53 (0.00959)	<0.001***	6676	8.53 (0.0121)	8.75 (0.0595)	8.52 (0.0118)	<0.001***
MET-IR	9605	41.87 (0.199)	44.72 (0.498)	41.59 (0.206)	<0.001***	6676	41.58 (0.204)	46.79 (1.52)	41.43 (0.205)	<0.001***
TG/HDL-C	9605	2.68 (0.0391)	2.97 (0.149)	2.65 (0.0403)	<0.001***	6676	2.73 (0.0504)	3.96 (0.611)	2.70 (0.0462)	0.0425*
HOMA-IR	9605	2.96 (0.0471)	3.43 (0.139)	2.91 (0.0467)	<0.001***	6676	2.96 (0.0569)	3.93 (0.322)	2.93 (0.0553)	0.002**
TyG-BMI index	9605	244.50 (1.06)	259.48 (2.68)	243.03 (1.09)	<0.001***	6676	242.13 (1.09)	268.42 (7.88)	241.36 (1.09)	<0.001***
SUA (mg/dl)	9605	5.45 (0.0199)	5.63 (0.0589)	5.43 (0.0207)	0.00130**	6676	5.47 (0.0254)	5.69 (0.120)	5.46 (0.0257)	0.0675
VD	9605	70.29 (0.669)	73.34 (1.52)	69.99 (0.698)	0.0358*	6675	69.24 (0.794)	72.42 (2.21)	69.14 (0.794)	0.125
Age, n (%)	9605				<0.001***	6676				0.00790**
Mean (SE)		45.7 (0.283)	51.1 (0.687)	45.1 (0.286)			45.6 (0.346)	50.1 (1.41)	45.4 (0.349)	
20–35		2908 (32.76%)	137 (18.35%)	2771 (34.17%)			2023 (32.43%)	28 (19.41%)	1995 (32.80%)	
35–65		4897 (52.88%)	486 (62.4%)	4411 (51.95%)			3401 (53.74%)	102 (60.71%)	3299 (53.54%)	
>65		1800 (14.36%)	206 (19.25%)	1594 (13.88%)			1252 (13.83%)	50 (19.88%)	1202 (13.66%)	
Gender, n (%)	9605				0.0149*	6676				0.0337*
Female		4948 (51.18%)	384 (46.58%)	4564 (51.63%)			3425 (50.85%)	74 (42.48%)	3351 (51.1%)	
Male		4657 (48.82%)	445 (53.42%)	4212 (48.37%)			3251 (49.15%)	106 (57.52%)	3145 (48.9%)	
Race, n (%)	9605				<0.001***	6676				<0.001***
Mexican American		1386 (7.96%)	95 (5.68%)	1291 (8.18%)			968 (7.81%)	16 (4.75%)	952 (7.9%)	
Non-Hispanic Black		1736 (9.92%)	93 (5.02%)	1643(10.4%)			1154 (9.94%)	10 (2.58%)	1144 (10.16%)	
Non-Hispanic White		4412 (69.56%)	488 (78.2%)	3924(68.72%)			3295 (71.2%)	124 (83.74%)	3171 (70.84%)	
Other Hispanic		959 (5.26%)	86 (5.16%)	873(5.27%)			635 (4.85%)	23 (5.67%)	612 (4.83%)	
Other Race		1112 (7.29%)	67 (5.94%)	1045(7.42%)			624 (6.19%)	7 (3.26%)	617 (6.28%)	
Education, n (%)	9605				0.354	6676				0.0223*
Below high school		1129 (8.48%)	108 (9.99%)	1021 (8.33%)			614 (7.34%)	17 (7.96%)	597 (7.32%)	
College or above		7129 (76.77%)	600 (74.56%)	6529 (76.99%)			5404 (83.05%)	139 (75.22%)	5265 (83.28%)	
High school		1347 (14.75%)	1226 (15.45%)	1226 (14.68%)			658 (9.62%)	24 (16.82%)	634 (9.4%)	
PIR, n (%)	9605				0.455	6676				0.190
PIR 1–3		3967 (35.62%)	326 (34.25%)	3641 (35.75%)			2679 (34.53%)	72 (40.59%)	2607 (34.35%)	
PIR < 1		1970 (14.25%)	168 (13.43%)	1802 (14.33%)			1387 (14.65%)	43 (16.41%)	1344 (14.6%)	
PIR > 3		3668 (50.13%)	335 (52.32%)	3333 (49.92%)			2610 (50.82%)	65 (43%)	2545 (51.05%)	
Smoke, n (%)	9605				0.704	6676				0.629
No		7638 (80.31%)	654 (79.74%)	6984 (80.36%)			5282 (79.74%)	138 (77.62%)	5144 (79.8%)	
Yes		1967 (19.69%)	175 (20.26%)	1792 (19.64%)			1394 (20.26%)	42 (22.38%)	1352 (20.2%)	
Drink, n (%)	9605				0.631	6676				0.450
No		2252 (18.37%)	192 (19.17%)	2060 (18.29%)			1697 (20.62%)	36 (23.65%)	1661 (20.53%)	
Yes		7353 (81.63%)	637 (80.83%)	6716 (81.71%)			4979 (79.38%)	144 (76.35%)	4835 (79.47%)	
Weight, n (%)	9605				<0.001***	6676				0.00316**
Normal		2873 (30.37%)	184 (21.58%)	2689 (31.23%)			2052 (31.67%)	33 (17.6%)	2019 (32.08%)	
Overweight		6560 (67.87%)	643 (78.16%)	5917 (66.86%)			4508 (66.72%)	147 (82.4%)	4361 (66.26%)	
Underweight		172 (1.76%)	2 (0.26%)	170 (1.91%)			116 (1.61%)	0	116 (1.66%)	
Marital, n (%)	9605				<0.001***	6676				0.005465**
Married		4900 (54.75%)	472 (63.13%)	4428 (53.92%)			3471 (56.03%)	103 (64.59%)	3368 (55.78%)	
Never married		1856 (19.25%)	83 (9.51%)	1773 (20.21%)			1280 (19.41%)	12 (7.32%)	1268 (19.76%)	
Other		2849 (26.00%)	274 (27.36%)	2575 (25.87%)			1925 (24.56%)	65 (28.09%)	1860 (24.45%)	
Hypertension, n %)	9605				<0.001***	6676				0.0196*
No		5989 (66.07%)	413(53.36%)	5576 (67.32%)			4172 (65.95%)	90(53.51%)	4082 (66.32%)	
Yes		3616 (33.93%)	416(46.64%)	3200 (32.68%)			2504 (34.05%)	90(46.49%)	2414 (33.68%)	

Continuous data are shown as mean (SE) and categorical data are shown as percentage. *: 0.01< =*p* < 0.05; **: 0.001< =*p* < 0.01; ***: *p* < 0.001.

An additional analysis of baseline characteristics was conducted for all participants across quartiles of insulin resistance indices (TyG index, TG/HDL-C, METs-IR, HOMA-IR and TyG-BMI) (Table S1–S5) revealed that age, gender, marital status, race, poverty-to-income ratio, weight status, education level, and hypertension were significantly associated with IR indices (*p* < 0.001). The high insulin resistance groups exhibited a higher proportion of older adults, males, non-Hispanic whites, overweight/obese individuals, and hypertensives. Additionally, the prevalence of kidney stones and their recurrence was significantly higher in these groups (*p* < 0.001).

### 3.2. Association of IR index with kidney stones and their recurrence

In this study, we developed three logistic regression models to comprehensively evaluate the association of five commonly used insulin resistance indices (TyG-BMI, METs-IR, HOMA-IR, TG/HDL-C, and TyG index) with kidney stones and their recurrence. After appropriate adjustment for covariates, our analyses revealed significant differences in the index. When the TyG index was used as a continuous variable, Model 1 showed that each unit increase was linked with a 31.9% increased risk of kidney stones (*p* < 0.001, OR = 1.319, 95% CI: 1.155–1.505). However, this association was attenuated and lost statistical significance in a further adjusted Model 2 (*p* = 0.09, OR = 1.131, 95% CI: 0.979–1.306). Hierarchical analysis showed that the highest quartile of TyG index (Q4) had a significantly higher risk of kidney stones in Model 1 (*p* < 0.001, OR = 1.700, 95% CI: 1.313–2.202) compared with Q1, but the association lost significance in Model 2. Similarly, TG/HDL-C demonstrated a significant association with kidney stones in the initial model, but the association lost significance after adjustment for covariates. In contrast, the results for METs-IR were more robust and statistically significant. Model 1 indicated that each unit increase in METs-IR was linked to a 2.0% rise in the risk of developing kidney stones (*p* < 0.001, OR = 1.020, 95% CI: 1.014–1.027), and this remained a significant positive correlation even in the fully adjusted Model 3 (*p* = 0.0073, OR = 1.012, 95% CI: 1.003–1.022). Hierarchical analyses further confirmed that the highest quartile group (Q4) exhibited a 2.421-fold greater risk of developing kidney stones in Model 1 (*p* < 0.001, OR = 2.421, 95% CI: 1.815–3.230) and remained significant in Model 3 (*p* = 0.0031, OR = 2.213, 95% CI: 1.320–3.711). HOMA- IR also showed a significant association, with a 5.1% increase in kidney stone risk per unit increase in HOMA-IR in Model 1 (*p* < 0.001, OR = 1.051, 95% CI: 1.025–1.078), which maintained statistical significance even in Model 3 (*p* = 0.02767). Finally, TyG-BMI, another key indicator of insulin resistance, had an OR of 1.523 (*p* = 0.001, 95% CI: 1.207–1.923) in Model 1, which remained statistically significant even in adjusted Model 2 (*p* = 0.030, OR = 1.241, 95% CI: 1.021–1.509). Hierarchical analyses showed that the highest quartile group (Q4) had a significantly higher risk of kidney stones by a factor of 2.274 (*p* < 0.001, OR = 2.274, 95% CI: 1.486–3.481), a result that was also supported in Model 3 (*p* = 0.0092, OR = 1.943, 95% CI: 1.176–3.211). Detailed data can be found in Table 2. In addition to this, we also found a strong association between HOMA-IR, METs-IR and TyG-BMI with kidney stone recurrence, and this association persisted after model adjustment (Table 3).

**Table 2.** Relationship between insulin resistance indices and kidney stones in the logistic regression models.

Kidney stones	Model 1		Model 2		Model 3	
	OR (95%Cl)	*P* Value	OR (95%Cl)	*P* Value	OR (95%Cl)	*P* Value
TyG index						
Continuous	1.319 (1.155–1.505)	<0.001***	1.131 (0.979–1.306)	0.0948	1.053 (0.905–1.226)	0.498
Q1	Reference		Reference		Reference	
Q2	1.415 (1.054–1.900)	0.0214*	1.219 (0.913–1.628)	0.177	1.081 (0.807–1.449)	0.598
Q3	1.527 (1.140–2.046)	0.00503**	1.242 (0.933–1.652)	0.136	1.030 (0.762–1.393)	0.843
Q4	1.700 (1.313–2.202)	<0.001***	1.304 (1.003–1.695)	0.0476*	1.005 (0.759–1.331)	0.970
P for trend	<0.001***		0.0717		0.869	
METs-IR						
Continuous	1.020 (1.014–1.027)	<0.001***	1.021 (1.014–1.028)	<0.001***	1.012 (1.003–1.022)	0.00730**
Q1	Reference		Reference		Reference	
Q2	1.515 (1.077–2.129)	0.0175*	1.406 (1.092–1.992)	0.0456*	1.358 (0.840–2.196)	0.208
Q3	2.278 (1.756–2.956)	<0.001***	2.071 (1.583–2.709)	<0.001***	2.019 (1.225–3.327)	0.00646**
Q4	2.421 (1.815–3.230)	<0.001***	2.312 (1.733–3.086)	<0.001***	2.213 (1.320–3.711)	0.00308**
P for trend	<0.001***		<0.001***		<0.001***	
TG/HDL-C						
Continuous	1.021 (1.006–1.036)	0.00539**	1.015 (0.997–1.033)	0.111	1.000 (0.976–1.024)	0.986
Q1	Reference		Reference		Reference	
Q2	1.026 (0.776–1.357)	0.854	0.962 (0.726–1.274)	0.783	0.849 (0.641–1.124)	0.249
Q3	1.434 (1.070–1.922)	0.0163*	1.285 (0.966–1.709)	0.0845	1.062 (0.787–1.432)	0.689
Q4	1.655 (1.263–2.170)	0.000374***	1.417 (1.082–1.857)	0.0119*	1.098 (0.825–1.459)	0.517
P for trend	<0.001***		0.00278**		0.238	
HOMA-IR						
Continuous	1.051 (1.025–1.078)	<0.001***	1.053 (1.027–1.079)	<0.001***	1.031 (1.003–1.059)	0.0277*
Q1	Reference		Reference		Reference	
Q2	1.243 (0.936–1.652)	0.132	1.242 (0.935–1.651)	0.133	1.112 (0.825–1.501)	0.480
Q3	1.787 (1.331–2.399)	<0.001***	1.758 (1.306–2.368)	<0.001***	1.452 (1.049–2.008)	0.0249*
Q4	1.857 (1.436–2.402)	<0.001***	1.797 (1.396–2.314)	<0.001***	1.402 (1.053–1.866)	0.0213*
P for trend	<0.001***		<0.001***		0.00709**	
TyG-BMI index						
Continuous	1.004 (1.003–1.005)	<0.001***	1.004 (1.002–1.005)	<0.001***	1.002 (1.001–1.004)	0.0272*
Q1	Reference		Reference		Reference	
Q2	1.439 (1.016–2.036)	0.0405*	1.269 (0.898–1.795)	0.175	1.054 (0.662–1.676)	0.823
Q3	2.128 (1.640–2.761)	<0.001***	1.835 (1.408–2.392)	<0.001***	1.425 (0.859–2.366)	0.167
Q4	2.135 (1.616–2.821)	<0.001***	1.944 (1.477–2.558)	<0.001***	1.459 (0.883–2.410)	0.138
P for trend	<0.001***		<0.001***		0.0335*	

Model 1 did not include covariates; Model 2 included three covariates of age, gender, and ethnicity, adjusting for basic information about the participants; Model 3 further incorporated additional personal information about the participants, namely, household income, PIR, education, and marital status. Additionally, it was adjusted for high-risk factors for kidney stone formation, including smoking, alcohol consumption, hypertension, and serum uric acid levels. *: 0.01< =*p* < 0.05; **: 0.001< =*p* < 0.01; ***: *p* < 0.001.

**Table 3.** Relationship between insulin resistance indices and kidney stone recurrence in the logistic regression models.

Kidney stone recurrence	Model 1		Model 2		Model 3	
	OR (95%Cl)	*P* Value	OR (95%Cl)	*P* Value	OR (95%Cl)	*P* Value
TyG index						
Continuous	1.789 (1.371–2.335)	<0.001***	1.575 (1.192–2.081)	0.00186**	1.308 (1.013–1.689)	0.0396*
Q1	Reference		Reference		Reference	
Q2	1.083 (0.543–2.159)	0.818	0.935 (0.471–1.856)	0.846	0.785 (0.382–1.615)	0.503
Q3	2.192 (1.074–4.477)	0.0317*	1.794 (0.873–3.686)	0.110	1.423 (0.672–3.016)	0.349
Q4	2.518 (1.429–4.440)	0.00184**	1.938 (1.106–3.395)	0.0217*	1.380 (0.803–2.372)	0.238
P for trend	<0.001***		0.00376**		0.0521	
METs-IR						
Continuous	1.033 (1.018–1.048)	<0.001***	1.034 (1.017–1.051)	<0.001***	1.024 (1.006–1.042)	<0.001***
Q1	Reference		Reference		Reference	
Q2	2.437 (1.196–4.967)	0.0151*	2.230 (1.095–4.540)	0.0278*	1.742 (0.716–4.240)	0.215
Q3	3.169 (1.413–7.107)	0.00587**	2.811 (1.198–6.593)	0.0184*	1.975 (0.626–6.231)	0.239
Q4	3.960 (1.867–8.398)	<0.001***	3.701 (1.696–8.076)	0.00143**	2.526 (0.889–7.179)	0.0806
P for trend	<0.001***		0.00281**		0.0252*	
TG/HDL-C						
Continuous	1.041 (1.024–1.059)	<0.001***	1.038 (1.019–1.058)	<0.001***	1.026 (1.004–1.048)	0.0201*
Q1	Reference		Reference		Reference	
Q2	0.660 (0.347–1.254)	0.201	0.598 (0.312–1.148)	0.120	0.490 (0.255–0.941)	0.0329*
Q3	1.651 (0.843–3.233)	0.141	1.454 (0.742–2.849)	0.270	1.113 (0.547–2.268)	0.762
Q4	2.531 (1.384–4.629)	0.00314**	2.102 (1.143–3.864)	0.0178*	1.446 (0.798–2.620)	0.217
P for trend	<0.001***		0.00142**		0.0208*	
HOMA-IR						
Continuous	1.079 (1.041–1.118)	<0.001***	1.081 (1.044–1.119)	<0.001***	1.057 (1.018–1.097)	0.00475**
Q1	Reference		Reference		Reference	
Q2	1.183 (0.522–2.708)	0.676	1.211 (0.532–2.756)	0.643	1.023 (0.447–2.342)	0.957
Q3	2.669 (1.307–5.447)	0.00782**	2.625 (1.286–5.360)	0.00897**	2.056 (1.002–4.220)	0.0495*
Q4	3.058 (1.373–6.812)	0.00701**	2.982 (1.342–6.629)	0.00826**	2.177 (1.048–4.520)	0.0375*
P for trend	<0.001***		<0.001***		0.00392**	
TyG-BMI index						
Continuous	1.006 (1.003–1.009)	<0.001***	1.006 (1.003–1.009)	<0.001***	1.004 (1.001–1.007)	0.0130*
Q1	Reference		Reference		Reference	
Q2	2.048 (1.029–4.078)	0.0416*	1.799 (0.896–3.612)	0.0972	1.499 (0.604–3.722)	0.375
Q3	3.257 (1.602–6.620)	0.00148**	2.789 (1.323–5.882)	0.00796**	2.173 (0.749–6.305)	0.149
Q4	3.646 (1.783–7.456)	<0.001***	3.334 (1.574–7.060)	0.00219**	2.514 (0.927–6.815)	0.0692
P for trend	<0.001***		0.00237**		0.0431*	

Model 1 did not include covariates; Model 2 included three covariates of age, gender, and ethnicity, adjusting for basic information about the participants; Model 3 further incorporated additional personal information about the participants, namely, household income, PIR, education, and marital status. Additionally, it was adjusted for high-risk factors for kidney stone formation, including smoking, alcohol consumption, hypertension, and serum uric acid levels. *: 0.01< =*p* < 0.05; **: 0.001< =*p* < 0.01; ***: *p* < 0.001.

### 3.3. Nonlinear analysis of IR index and kidney stones as well as their recurrence

**Figure 2.**
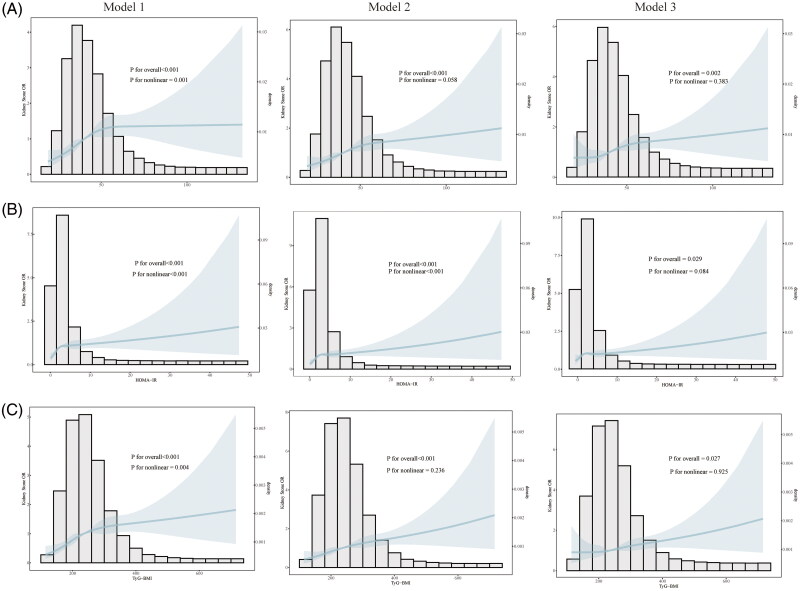
After correcting for covariates, the associations between METs-IR (Figure 2A), HOMA-IR (Figure 2B), and TyG-BMI (Figure 2C) and the occurrence of kidney stones were assessed by RCS curves. The solid blue line corresponds to the Central estimate and the light blue shaded area indicates the 95% confidence interval. Model 1 did not include covariates; Model 2 included three covariates of age, gender, and ethnicity, adjusting for basic information about the participants; Model 3 further incorporated additional personal information about the participants, namely, household income, PIR, education, and marital status. Additionally, it was adjusted for high-risk factors for kidney stone formation, including smoking, alcohol consumption, hypertension, and serum uric acid levels.

Considering that the weighted logistic regression analysis revealed a strong correlation between METs-IR, HOMA-IR, and TyG-BMI and kidney stones as well as kidney stone recurrence; to investigate the nonlinear association between the above three IR indices and kidney stones as well as kidney stone recurrence in depth, we investigated the correlation using RCS analysis. Figure 2 demonstrated the relationship between the three IR indices and kidney stones, and the results suggested that the incidence of kidney stones progressively increase as the IR indices increase. For METs-IR (Figure 2(A)), in unadjusted Model 1, each incremental unit in METs-IR was linked to a gradual elevation in the risk of kidney stones, exhibiting a notable nonlinear pattern (P for nonlinearity = 0.001). However, in fully adjusted Model 3, this nonlinear trend changed to a linear trend (P for nonlinearity = 0.383), suggesting that METs-IR has a complex relationship with the risk of kidney stones, but is positively correlated overall. For HOMA-IR (Figure 2(B)), Model 1 also showed a significant nonlinear association (P for nonlinearity < 0.001), and this trend remained statistically significant in adjusted Model 3 (P for nonlinearity = 0.084), but with diminished strength. TyG-BMI (Figure 2(C)), on the other hand, showed the same significant nonlinear trend in unadjusted Model 1 (P for nonlinearity = 0.004), but the nonlinear association disappeared after adjustment in Model 3 (P for nonlinearity = 0.925), suggesting that the effect of TyG-BMI was more linear after full adjustment. Furthermore, Figure S1 showed a linear association between the three IR indices and recurrence in the fully adjusted models, suggesting that increasing IR indices are strongly associated with increased risk of recurrence.

### 3.4. Mediation analysis

**Figure 3.**
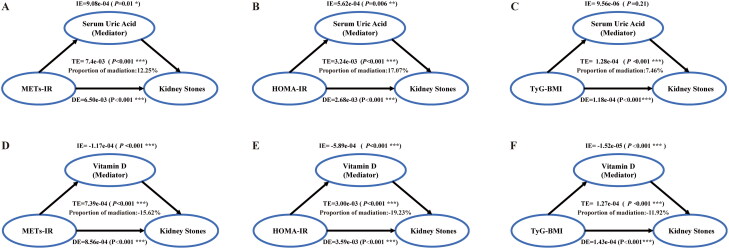
Mediation analysis of the interaction between serum uric acid and vitamin D levels on three indices of insulin resistance and the risk of kidney stones.

Mediation analysis showed that serum uric acid and vitamin D played different mediating roles between IR indices (HOMA-IR, METs-IR and TyG-BMI) and kidney stone occurrence. As shown in Figure 3, serum uric acid demonstrated a notable positive mediating effect between METs-IR and HOMA-IR and kidney stones(12.25% and 17.07% of the total effect, respectively), but a nonsignificant mediating effect between TyG-BMI and kidney stones (*p* = 0.16). Vitamin D, on the other hand, showed a significant negative mediating effect (*p* < 0.001) between all three IR indices and kidney stones, with a significant indirect effect on METs-IR and HOMA-IR (19.23% and 15.62% of the total effect, respectively), and a significant indirect effect on TyG-BMI (11.92% of the total effect). The roles of serum uric acid and vitamin D in the three IR indices and kidney stone recurrence were further explored. The results, as shown in Figure S2, serum uric acid demonstrated a notable mediating effect (mediation ratio of 14.22%, *p* = 0.011) on the association between HOMA-IR and the recurrence of kidney stones, while no such effect was observed for METs-IR and TyG-BMI. In contrast, vitamin D showed a negative mediating effect in all IR indices, especially in HOMA-IR with a mediating proportion of 17.76%.

### 3.5. Predictive power of the IR index for kidney stones and their recurrence

**Figure 4.**
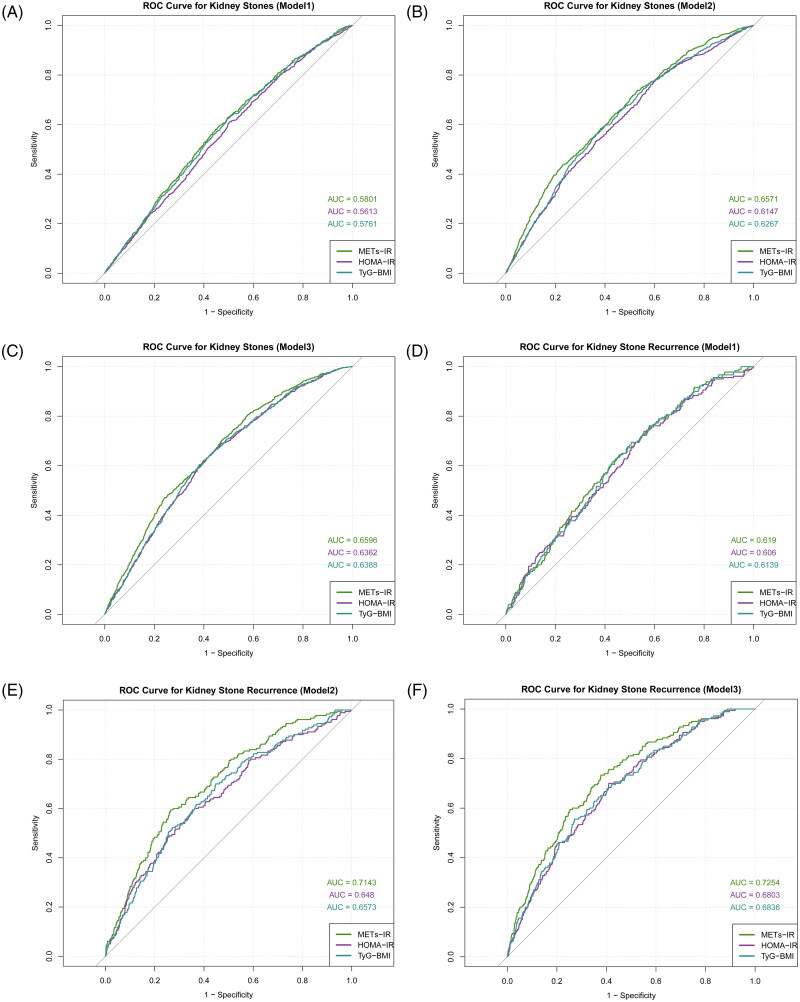
Predictive effectiveness of IR indices on the incidence of kidney stones (Figure 4A–C) and their recurrence (Figure 4D–F) in three models.

Figures 4(A–C) illustrate the predictive performance of different IR indices for kidney stone incidence. Across all three models, after adjusting for various covariates, METs-IR consistently achieved a higher area under the curve (AUC) than HOMA-IR and TyG-BMI, highlighting its superior predictive capability. Meanwhile, Figures 4(D–F) demonstrate the prognostic value of IR indices for kidney stone recurrence. Notably, in Model 2 and Model 3, the AUC of METs-IR exceeded 0.7, indicating its strong predictive advantage when combined with personal information and other risk factors.

### 3.6. Subgroup analysis

**Figure 5.**
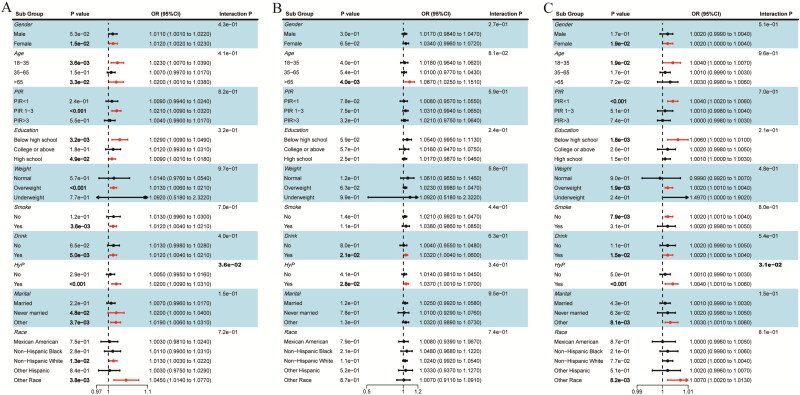
Subgroup analysis of the associations between insulin resistance indices (METs-IR(**A**), HOMA-IR(**B**) and TyG-BMI(**C**)) and kidney stones. Adjusted for age, gender, race, weight, PIR, education, drink, smoke, CVD, uric acid, marital status and hypertension, except the subgroup factors themselves.

In order to further explore the effects of the three indices of insulin resistance on the outcome measures, we performed subgroup analyses of all the covariates. Figure 5 demonstrated the subgroup analysis of the association between the three IR indices and kidney stones, in which there was no significant interaction except for the hypertension subgroup (METs-IR: interaction *p* = 0.036; TyG-BMI: interaction *p* = 0.031), in which both METs-IR and TyG-BMI were strongly associated with the prevalence of kidney stones in hypertensive patients (*p* < 0.001, OR = 1.02, 95%CI: 1.009–1.031; *p* < 0.001, OR = 1.004, 95% Cl: 1.001–1.007), whereas in non-hypertensive patients the association was not significant. Subgroup analyses of the correlation between the three IR indices and recurrence of kidney stones were shown in Figure S3, and the results revealed no notable interactions across any of the subgroups, suggesting that the correlation holds across a wide range of demographic situations.

## 4. Discussion

In this cross-sectional study, which included 9,605 non-diabetic patients, we conducted a systematic evaluation of the association between five indices of IR (METs-IR, HOMA-IR, TyG index, TG/HDL-C and TyG-BMI) and kidney stones as well as their recurrence for the first time. The results demonstrated that METs-IR, HOMA-IR, and TyG-BMI were all notably and positively associated with the risk of kidney stones and their recurrence. This positive association was consistent across all covariate-stratified subgroups. Mediation analyses indicated that vitamin D and serum uric acid exerted disparate mediating effects in this association, suggesting that these biomarkers may play a key position in the pathogenesis of insulin resistance and kidney stones. This finding provided important clues for further mechanistic studies and early clinical intervention. Furthermore, it is noteworthy that METs-IR, a validated indicator of insulin resistance, exhibited a higher AUC than HOMA-IR and TyG-BMI, thereby demonstrating a certain predictive ability for the occurrence and recurrence of kidney stones. This finding needs to be validated by subsequent prospective studies.

IR is a core indicator of metabolic syndrome, which refers to the body’s reduced sensitivity to insulin, which triggers a series of metabolic abnormalities [29]. Although the hyper-insulinemic euglycemic clamp (HINC) test is recognized internationally as the gold standard for evaluating IR, its complexity and high cost have limited its clinical application [30]. Nowadays, the commonly used clinical IR indices, such as METs-IR, TG/HDL-C, HOMA-IR, and TyG index, as well as its expanded indicator TyG-BMI, have been proved to be representative in IR assessment [31,32], and have high clinical utility due to their simple calculation and easy accessibility. In recent years, a growing body of research has demonstrated the excellent predictive power of the IR indices in a wide range of diseases. Duan et al. find strong benefits of METs-IR in predicting cardiovascular disease [14], Shen et al. find that METs-IR and TyG-BMI were strongly associated with an elevated risk of kidney stones [33]. Shi et al. on the other hand, found that the insulin resistance index possessed stronger predictive power in patients with NAFLD compared to indicators of obesity [34]. Additionally, Qiu et al. discovered a notable positive correlation between TyG index and hyperuricemia in U.S. adults [35]. These findings indirectly support the positive correlation between the IR indices and the incidence and recurrence of kidney stones. Moreover, previous large-scale cross-sectional studies have demonstrated the predictive capability of the IR indices regarding kidney stones [33,36–38]. However, these studies were not discussed separately for non-diabetic patients. While diabetic patients are typically associated with a range of metabolic disorders [39,40], these factors may complicate the direct correlation between IR and kidney stones. Focusing on non-diabetic patients and including a study population with recurrent kidney stones, the present study systematically investigated the relationship between IR indices and kidney stones as well as their recurrence. This study offers significant insights into the mechanism of IR in kidney stones formation, eliminates diabetes-related confounding factors, and enables a more accurate assessment of the independent effect of IR on kidney stones risk.

Kidney stone is a very complex chronic disease, and previous studies have demonstrated that IR may promote the formation and recurrence of renal stones through multiple metabolic mechanisms. First, IR is closely associated with urinary acidification [41], and an acidic urinary environment not only promotes the formation of uric acid stones, but may also accelerate the deposition of calcium oxalate crystals [42]. It has been demonstrated that IR therapy results in an elevation of renal ammonium nitrogen excretion, which in turn produces an increase in urinary pH [43]. Furthermore, IR reduces urinary citrate excretion, and since citrate is a key crystallization inhibitor, its reduction increases the risk of kidney stone formation [44,45]. As IR worsens and insulin levels remain persistently elevated, this disruption in citrate metabolism may further contribute to stone formation by promoting an environment conducive to crystallization. Not only that, other metabolic syndrome components, including obesity and hypertension, significantly contribute to the mechanisms behind kidney stone formation. Obesity affects vitamin D metabolism and induces chronic inflammation, which can disrupt calcium-phosphate homeostasis and contribute to kidney stone formation [46,47], while hypertension further increases the risk of kidney stones by decreasing urinary citrate levels [48]. Mediation analysis revealed that serum uric acid and vitamin D play distinct mediating roles in the relationship between IR indices and kidney stones risk. Serum uric acid not only increases the risk of uric acid stone formation by promoting urinary acidification but may also contribute to calcium stone formation by inducing renal tubular cell damage and calcium deposition [49]. In contrast, vitamin D plays a key role in maintaining calcium-phosphorus homeostasis, and its deficiency may mediate the relationship between IR indices and kidney stone risk by regulating urinary calcium levels and aggravating inflammation associated with IR [50]. However, even after adjusting for covariates such as smoking, BMI and hypertension, we still discovered that the correlation between IR index and kidney stones persisted, suggesting that there is an independent role of insulin resistance index on the occurrence as well as recurrence of kidney stones.

The present study conducted a weighted analysis on a large sample of 9,605 non-diabetic patients to investigate the relationship between five IR surrogates and the incidence and recurrence of kidney stones. Indicators with significant positive correlations were then selected for further in-depth statistical analysis. By focusing for the first time on the non-diabetic population, we were able to effectively eliminate diabetes-related confounders and thus more accurately assess the independent effect of insulin resistance on kidney stone risk. The results of this study enhance our understanding of the predictive value of IR for kidney stones and recurrence in the non-diabetic population, aiding in the identification of high-risk patients and the development of personalized prevention and treatment strategies. Furthermore, our findings indicate that serum uric acid and vitamin D partially mediate the relationship between IR indices and the increased risk of kidney stones and their recurrence, offering valuable new insights for early screening and intervention in kidney stone management. However, it is undeniable that this study still has certain limitations. First, as a retrospective study based on the NHANES database, the results of this study necessitate further prospective research to investigate the causal relationship between IR indices and the occurrence and recurrence of kidney stones. Second, because of the limitations of NHANES, the determination of kidney stone and recurrence was based on patient self-report, and recall bias could not be avoided. Furthermore, as this study focused on a U.S. population, extrapolation of the results is limited, and subsequent studies should be validated in multiple centers to increase the generalizability and reliability of the results. Finally, although the NHANES dataset contains a wealth of metabolic-related information, the cross-sectional nature of the data limits our ability to explore the temporal relationship or dynamic changes between IR and kidney stone formation. Additionally, selection bias may be present, potentially affecting the generalizability of the analysis. Therefore, future studies should adopt a multi-center design and incorporate long-term follow-up data to further validate these findings and explore the mechanisms by which IR, serum uric acid, vitamin D, and other factors contribute to kidney stones formation and recurrence.

## 5. Conclusion

In this study, we evaluated the association of IR indices (TyG-BMI, METs-IR, HOMA-IR) with kidney stones and their recurrence in a non-diabetic population and found that elevated insulin resistance indices significantly increased the risk of kidney stones. Vitamin D and serum uric acid played different mediating roles in this association, with METs-IR performing best in predicting incident and recurrent kidney stones. This study provides new insights into the mechanisms of insulin resistance in kidney stone formation and highlights the importance of individualized risk assessment.

## Supplementary Material

Supplemental files.docx

## Data Availability

All data can be obtained from the online NHANES website: https://www.cdc.gov/nchs/nhanes/index.htm.
