# Supplementary material for: Association of insulin resistance indices with kidney stones and their recurrence in a non-diabetic population: an analysis based on NHANES data from 2007–2018
Source: Ren Fail. 2025 Apr 24;47(1):2490203. doi: 10.1080/0886022X.2025.2490203 (PMC12035944; doi:10.1080/0886022X.2025.2490203)
Supplement: Supplemental files.docx [file IRNF_A_2490203_SM8379.docx]

**Figure Legends：**

**Figure S1**：After correcting for covariates, the associations between METs-IR (**Figure S1A**), HOMA-IR (**Figure S1B**), and TyG-BMI (**Figure S1C**) and the occurrence of kidney stone recurrence were assessed by RCS curves. The solid blue line corresponds to the central estimate and the light blue shaded area indicates the 95% confidence interval. Model 1 did not include covariates; Model 2 included three covariates of age, gender, and ethnicity, adjusting for basic information about the participants; Model 3 further incorporated additional personal information about the participants, namely, household income, PIR, education, and marital status. Additionally, it was adjusted for high-risk factors for kidney stone formation, including smoking, alcohol consumption, hypertension, and serum uric acid levels.

**Figure S2**: Mediation analysis of the interaction between serum uric acid and vitamin D levels on three indices of insulin resistance and the risk of kidney stone recurrence.

**Figure S3**: Subgroup analysis of the association between insulin resistance indices (METs-IR(**A**), HOMA-IR(**B**) and TyG-BMI(**C**)) and kidney stones recurrence. Adjusted for age, gender, race, Weight, PIR, education, drink, smoke, CVD, uric acid, marital status and hypertension, except the subgroup factors themselves.

**Table S1**：Baseline characteristics according to TyG index quartiles.

| Characteristic | Overall, N | TyG index levels | | | | | |
| --- | --- | --- | --- | --- | --- | --- | --- |
|  |  | Overall | Q1 | Q2 | Q3 | Q4 | P |
| TyG index | 9605 | 8.54（0.00910） | 7.83 (0.00711) | 8.32 (0.00284) | 8.70 (0.00328) | 9.31 (0.00989) | <0.001 |
| MET-IR | 9605 | 41.9（0.199） | 34.78 (0.237) | 39.24 (0.296) | 43.34 (0.339) | 50.14 (0.344) | <0.001 |
| TG/HDL-C | 9605 | 2.68（0.0391） | 0.93 (0.0103) | 1.55 (0.0139) | 2.43 (0.0204) | 5.8 (0.118) | <0.001 |
| HOMA-IR | 9605 | 2.96（0.0471） | 1.74 (0.0400) | 2.48 (0.0620) | 3.18 (0.0754) | 4.44 (0.121) | <0.001 |
| TyG-BMI index | 9605 | 244.50（1.06） | 203.09 (1.23) | 231.79 (1.58) | 255.10 (1.79) | 288.03 (1.75) | <0.001 |
| SUA（mg/dl） | 9605 | 5.45（0.0199） | 4.85 (0.0292) | 5.26 (0.0339) | 5.64 (0.0408) | 6.04 (0.0369) | <0.001 |
| VD3 | 9605 | 70.29（0.669） | 69.68 (1.04) | 70.57 (1.00) | 71.80 (1.03) | 69.11 (0.93) | 0.454 |
| Age, n（%） | 9605 |  |  |  |  |  | <0.001 |
| 20-35 |  | 2908 (32.76%) | 1086 (47.37%) | 719 (32.77%) | 602 (28.81%) | 501 (22.08%) |  |
| 35-65 |  | 4897 (52.88%) | 1035 (44.52%) | 1186 (52.01%) | 1245 (53.74%) | 1431 (61.24%) |  |
| >65 |  | 1800 (14.36%) | 274 (8.11%) | 484 (15.22%) | 516 (17.45%) | 526 (16.67%) |  |
| Gender, n (%) | 9605 |  |  |  |  |  | <0.001 |
| Female |  | 4948 (51.18%) | 1468 (61.61%) | 1273 (53.65%) | 1136 (46.98%) | 1071 (42.48%) |  |
| male |  | 4657 (48.82%) | 927 (38.39%) | 1116 (46.35%) | 1227 (53.02%) | 1387 (57.52%) |  |
| Race, n（%） | 9605 |  |  |  |  |  | <0.001 |
| Mexican American |  | 1386 (7.96%) | 236 (6.10%) | 298 (6.85%) | 401 (9.05%) | 451 (9.84%) |  |
| Non-Hispanic Black |  | 1736 (9.92%) | 713 (17.24%) | 508 (11.38%) | 314 (6.63%) | 201 (4.44%) |  |
| Non-Hispanic White |  | 4412 (69.56%) | 954 (64.26%) | 1099 (70.18%) | 1119 (71.05%) | 1240 (72.77%) |  |
| Other Hispanic |  | 959 (5.26%) | 202 (5.10%) | 219 (4.86%) | 249 (5.47%) | 289 (5.62%) |  |
| Other Race |  | 1112 (7.29%) | 290 (7.30%) | 265 (6.73%) | 280 (7.80%) | 277 (7.33%) |  |
| Education, n（%） | 9605 |  |  |  |  |  | <0.001 |
| Below high school |  | 1129 (8.48%) | 219 (6.82%) | 289 (8.56%) | 277 (8.71%) | 344 (9.83%) |  |
| College or above |  | 7129 (76.77%) | 1848 (79.83%) | 1808 (78.47%) | 1713 (74.94%) | 1760 (73.84%) |  |
| High school |  | 1347 (14.75%) | 328 (13.35%) | 292 (12.97%) | 373 (16.35%) | 354 (16.33%) |  |
| PIR, n（%） | 9605 |  |  |  |  |  | 0.01852 |
| PIR 1-3 |  | 3967 (35.62%) | 949 (32.98%) | 963 (34.30%) | 969 (36.27%) | 1086 (38.92%) |  |
| PIR<1 |  | 1970 (14.25%) | 473 (14.56%) | 476 (13.65%) | 495 (14.74%) | 526 (14.05%) |  |
| PIR>3 |  | 3668 (50.13%) | 973 (52.46%) | 950 (52.05%) | 899 (48.98%) | 846 (47.03%) |  |
| Smoke, n（%） | 9605 |  |  |  |  |  | <0.001 |
| No |  | 7638 (80.31%) | 2006 (84.05%) | 1908 (80.86%) | 1859 (79.48%) | 1865 (76.83%) |  |
| Yes |  | 1967 (19.69%) | 389 (15.95%) | 481 (19.14%) | 504 (20.52%) | 593 (23.17%) |  |
| Drink, n（%） | 9605 |  |  |  |  |  | 0.02683 |
| No |  | 2252 (18.37%) | 580 (19.2%) | 611 (20.32%) | 520 (16.87%) | 541 (17.08%) |  |
| Yes |  | 7353 (81.63%) | 1815 (80.8%) | 1778 (79.68%) | 1843 (83.13%) | 1917 (82.92%) |  |
| Weight, n（%） | 9605 |  |  |  |  |  | <0.001 |
| Normal |  | 2873 (30.37%) | 1094 (48.98%) | 813 (34.24%) | 603 (24.89%) | 363 (13.37%) |  |
| Overweight |  | 6560 (67.87%) | 1219 (47.64%) | 1519 (63.61%) | 1739 (74.24%) | 2083 (86.01%) |  |
| Underweight |  | 172 (1.76%) | 82 (3.38%) | 57 (2.15%) | 21 (0.87%) | 12 (0.62%) |  |
| Marital, n（%） | 9605 |  |  |  |  |  | <0.001 |
| Married |  | 4900 (54.75%) | 1076 (50.02%) | 1208 (55.86%) | 1244 (54.02%) | 1372 (59.08%) |  |
| Never married |  | 1856 (19.25%) | 687 (26.43%) | 476 (19.49%) | 385 (17.56%) | 308 (13.54%) |  |
| Other |  | 2849 (26%) | 632 (23.55%) | 705 (24.65%) | 734 (28.42%) | 778 (27.38%) |  |
| Hypertension, n（%） | 9605 |  |  |  |  |  | <0.001 |
| No |  | 5989 (66.07%) | 1816 (81.07%) | 1518 (67.72%) | 1378 (60.95%) | 1277 (54.54%) |  |
| Yes |  | 3616 (33.93%) | 579 (18.93%) | 871 (32.28%) | 985 (39.05%) | 1181 (45.46%) |  |
| Kidney Stones, n（%） | 9605 |  |  |  |  |  | 0.00188 |
| No |  | 8776 (91.07%) | 2247 (93.48%) | 2188 (91.02%) | 2142 (90.38%) | 2199 (89.4%) |  |
| Yes |  | 829 (8.93%) | 148 (6.52%) | 201 (8.98%) | 221 (9.62%) | 259 (10.6%) |  |
| Kidney Stone recurrence, n（%） | 6676 |  |  |  |  |  | 0.001735 |
| Yes |  | 180 (2.84%) | 28（1.71%） | 32（1.85%） | 51（3.68%） | 69（4.2%） |  |
| No |  | 6496 (97.16%) | 1690（98.29%） | 1639（98.15%） | 1554（96.32%） | 1613（95.8%） |  |

Quartile 1: TyG<=8.13; Quartile 2: 8.13<TyG<=8.51; Quartile 3: 8.51<TyG<=8.92; Quartile 4: 8.92<TyG

Continuous data are shown as mean (SE) and categorical data are shown as percentage.

*: 0.01<=P<0.05; **: 0.001<=P<0.01; ***: P<0.001

**Table S2**：Baseline characteristics according to METs-IR quartiles.

| Characteristic | Overall, N | METs-IR levels | | | | | |
| --- | --- | --- | --- | --- | --- | --- | --- |
|  |  | Overall | Q1 | Q2 | Q3 | Q4 | P |
| TyG index | 9605 | 8.54（0.00910） | 8.12 (0.0123) | 8.43 (0.0129) | 8.69 (0.0153) | 8.93 (0.0198) | <0.001*** |
| MET-IR | 9605 | 41.9（0.199） | 29.00 (0.0795) | 36.60 (0.0490) | 43.94 (0.0607) | 57.95 (0.282) | <0.001*** |
| TG/HDL-C | 9605 | 2.68（0.0391） | 1.22 (0.0197) | 1.94 (0.0300) | 2.93 (0.0603) | 4.64 (0.132) | <0.001*** |
| HOMA-IR | 9605 | 2.96（0.0471） | 1.44 (0.0303) | 2.12 (0.0390) | 3.02 (0.0482) | 5.25 (0.114) | <0.001*** |
| TyG-BMI index | 9605 | 244.50（1.06） | 177.06 (0.583) | 217.82 (0.504) | 255.31 (0.479) | 327.8 (1.57) | <0.001*** |
| SUA（mg/dl） | 9605 | 5.45（0.0199） | 4.72 (0.0343) | 5.25 (0.0358) | 5.7 (0.0320) | 6.12 (0.0337) | 0.00213** |
| VD3 | 9605 | 70.29（0.669） | 76.64 (1.06) | 73.92 (0.810) | 68.47 (1.01) | 62.18 (0.928) | 0.025* |
| Age, n（%） | 9605 |  |  |  |  |  | <0.001*** |
| 20-35 |  | 2908 (32.76%) | 905 (41.12%) | 688 (31.37%) | 597 (26.69%) | 718 (31.85%) |  |
| 35-65 |  | 4897 (52.88%) | 985 (45.19%) | 1213 (51.46%) | 1343 (57.58%) | 1356 (57.28%) |  |
| >65 |  | 1800 (14.36%) | 413 (13.69%) | 533 (17.16%) | 526 (15.73%) | 328 (10.87%) |  |
| Gender, n (%) | 9605 |  |  |  |  |  | <0.001*** |
| Female |  | 4948 (51.18%) | 1461 (66.24%) | 1198 (49.06%) | 1091 (42.32%) | 1198 (47.09%) |  |
| male |  | 4657 (48.82%) | 842 (33.76%) | 1236 (50.94%) | 1375 (57.68%) | 1204 (52.91%) |  |
| Race, n（%） | 9605 |  |  |  |  |  | <0.001*** |
| Mexican American |  | 1386 (7.96%) | 191 (4.60%) | 315 (6.92%) | 452 (10.22%) | 428 (10.10%) |  |
| Non-Hispanic Black |  | 1736 (9.92%) | 391 (9.30%) | 442 (9.97%) | 449 (9.74%) | 454 (10.68%) |  |
| Non-Hispanic White |  | 4412 (69.56%) | 1101 (71.26%) | 1128 (70.28%) | 1069 (68.24%) | 1114 (68.48%) |  |
| Other Hispanic |  | 959 (5.26%) | 178 (4.38%) | 240 (4.74%) | 289 (6.27%) | 252 (5.66%) |  |
| Other Race |  | 1112 (7.29%) | 442 (10.47%) | 309 (8.09%) | 207 (5.53%) | 154 (5.08%) |  |
| Education, n（%） | 9605 |  |  |  |  |  | 0.001549** |
| Below high school |  | 1129 (8.48%) | 211 (6.73%) | 293 (8.52%) | 313 (9.23%) | 312 (9.44%) |  |
| College or above |  | 7129 (76.77%) | 1793 (81.05%) | 1832 (77.24%) | 1785 (74.85%) | 1719 (73.95%) |  |
| High school |  | 1347 (14.75%) | 299 (12.23%) | 309 (14.23%) | 368 (15.93%) | 371 (16.60%) |  |
| PIR, n（%） | 9605 |  |  |  |  |  | <0.001*** |
| PIR 1-3 |  | 3967 (35.62%) | 949 (32.13%) | 963 (35.11%) | 969 (36.40%) | 1086 (38.84%) |  |
| PIR<1 |  | 1970 (14.25%) | 473 (13.91%) | 476 (12.81%) | 495 (13.80%) | 526 (16.48%) |  |
| PIR>3 |  | 3668 (50.13%) | 973 (53.96%) | 950 (52.08%) | 899 (49.80%) | 846 (44.68%) |  |
| Smoke, n（%） | 9605 |  |  |  |  |  | 0.3482 |
| No |  | 7638 (80.31%) | 1812 (79.38%) | 1934 (79.34%) | 2000 (80.94%) | 1892 (81.56%) |  |
| Yes |  | 1967 (19.69%) | 491 (20.62%) | 500 (20.66%) | 466 (19.06%) | 510 (18.44%) |  |
| Drink, n（%） | 9605 |  |  |  |  |  | 0.6572 |
| No |  | 2252 (18.37%) | 553 (18.36%) | 557 (17.63%) | 569 (18.24%) | 573 (19.25%) |  |
| Yes |  | 7353 (81.63%) | 1750 (81.64%) | 1877 (82.37%) | 1897 (81.76%) | 1829 (80.75%) |  |
| Weight, n（%） | 9605 |  |  |  |  |  | <0.001*** |
| Normal |  | 2873 (30.37%) | 1994 (86.54%) | 815 (33.14%) | 63 (1.79%) | 1 (0.01%) |  |
| Overweight |  | 6560 (67.87%) | 137 (6.43%) | 1619 (66.86%) | 2403 (98.21%) | 2401 (99.99%) |  |
| Underweight |  | 172 (1.76%) | 172 (7.03%) | 0 | 0 | 0 |  |
| Marital, n（%） | 9605 |  |  |  |  |  | <0.001*** |
| Married |  | 4900 (54.75%) | 1064 (50.89%) | 1229 (53.68%) | 1363 (58.29%) | 1244 (56.11%) |  |
| Never married |  | 1856 (19.25%) | 622 (25.53%) | 432 (18.04%) | 368 (15.54%) | 434 (17.91%) |  |
| Other |  | 2849 (26%) | 617 (23.58%) | 773 (28.28%) | 735 (26.17%) | 724 (25.98%) |  |
| Hypertension, n（%） | 9605 |  |  |  |  |  | <0.001*** |
| No |  | 5989 (66.07%) | 1743 (80.39%) | 1594 (68.8%) | 1396 (60.51%) | 1256 (54.58%) |  |
| Yes |  | 3616 (33.93%) | 560 (19.61%) | 840 (31.2%) | 1070 (39.49%) | 1146 (45.42%) |  |
| Kidney Stones, n（%） | 9605 |  |  |  |  |  | <0.001*** |
| No |  | 8776 (91.07%) | 2177 (94.8%) | 2251 (92.33%) | 2218 (88.89%) | 2130 (88.27%) |  |
| Yes |  | 829 (8.93%) | 126 (5.2%) | 183 (7.67%) | 248 (11.11%) | 272 (11.73%) |  |
| Kidney Stone recurrence, n（%） | 6676 |  |  |  |  |  | <0.001*** |
| Yes |  | 180 (2.84%) | 19（1.11%） | 40（2.66%） | 56（3.43%） | 65（4.25%） |  |
| No |  | 6496 (97.16%) | 1605（98.89%） | 1691（97.34%） | 1660（96.57%） | 1540（95.75%） |  |

Quartile 1: METs-IR<=33.20; Quartile 2: 33.20<METs-IR<=40.07; Quartile 3: 40.07<METs-IR<=48.27; Quartile 4: 48.27<METs-IR

Continuous data are shown as mean (SE) and categorical data are shown as percentage.

*: 0.01<=P<0.05; **: 0.001<=P<0.01; ***: P<0.001

**Table S3**：Baseline characteristics according to TG/HDL-C quartiles.

| Characteristic | Overall, N | TG/HDL-C levels | | | | | |
| --- | --- | --- | --- | --- | --- | --- | --- |
|  |  | Overall | Q1 | Q2 | Q3 | Q4 | P |
| TyG index | 9605 | 8.54（0.00910） | 7.89 (0.00934) | 8.33 (0.00633) | 8.68 (0.00723) | 9.27 (0.0114) | <0.001*** |
| MET-IR | 9605 | 41.9（0.199） | 33.38 (0.231) | 39.3 (0.326) | 43.76 (0.282) | 51.05 (0.327) | <0.001*** |
| TG/HDL-C | 9605 | 2.68（0.0391） | 0.84 (0.00618) | 1.5 (0.00502) | 2.42 (0.00947) | 5.97 (0.115) | <0.001*** |
| HOMA-IR | 9605 | 2.96（0.0471） | 1.74 (0.0410) | 2.5 (0.0658) | 3.14 (0.0657) | 4.46 (0.115) | <0.001*** |
| TyG-BMI index | 9605 | 244.50（1.06） | 201.48 (1.30) | 233.17 (1.85) | 255.75 (1.60) | 287.56 (1.68) | <0.001*** |
| SUA（mg/dl） | 9605 | 5.45（0.0199） | 4.81 (0.0298) | 5.24 (0.0335) | 5.62 (0.0350) | 6.12 (0.0390) | <0.001*** |
| VD3 | 9605 | 70.29（0.669） | 72.87 (1.06) | 70.76 (0.982) | 70.95 (0.896) | 66.54 (0.820) | 0.068 |
| Age, n（%） | 9605 |  |  |  |  |  | <0.001*** |
| 20-35 |  | 2908 (32.76%) | 862 (38.27%) | 758 (34.33%) | 668 (30.61%) | 620 (27.83%) |  |
| 35-65 |  | 4897 (52.88%) | 1122 (47.85%) | 1165 (51.33%) | 1224 (53.3%) | 1386 (59.03%) |  |
| >65 |  | 1800 (14.36%) | 417 (13.87%) | 471 (14.34%) | 503 (16.09%) | 409 (13.14%) |  |
| Gender, n (%) | 9605 |  |  |  |  |  | <0.001*** |
| Female |  | 4948 (51.18%) | 1574 (66.35%) | 1324 (56.27%) | 1147 (46.56%) | 903 (35.55%) |  |
| male |  | 4657 (48.82%) | 827 (33.65%) | 1070 (43.73%) | 1248 (53.44%) | 1512 (64.45%) |  |
| Race, n（%） | 9605 |  |  |  |  |  | <0.001*** |
| Mexican American |  | 1386 (7.96%) | 226 (5.59%) | 304 (7.02%) | 414 (9.09%) | 442 (10.13%) |  |
| Non-Hispanic Black |  | 1736 (9.92%) | 678 (15.91%) | 504 (11.42%) | 354 (7.79%) | 200 (4.57%) |  |
| Non-Hispanic White |  | 4412 (69.56%) | 999 (66.48%) | 1095 (70.19%) | 1107 (69.86%) | 1211 (71.73%) |  |
| Other Hispanic |  | 959 (5.26%) | 191 (4.53%) | 233 (5.00%) | 244 (5.38%) | 291 (6.13%) |  |
| Other Race |  | 1112 (7.29%) | 307 (7.49%) | 258 (6.36%) | 276 (7.88%) | 271 (7.43%) |  |
| Education, n（%） | 9605 |  |  |  |  |  | <0.001*** |
| Below high school |  | 1129 (8.48%) | 219 (6.35%) | 273 (8.3%) | 303 (8.97%) | 334 (10.31%) |  |
| College or above |  | 7129 (76.77%) | 1876 (81.18%) | 1800 (77.54%) | 1725 (74.79%) | 1728 (73.58%) |  |
| High school |  | 1347 (14.75%) | 306 (12.47%) | 321 (14.15%) | 367 (16.24%) | 353 (16.11%) |  |
| PIR, n（%） | 9605 |  |  |  |  |  | <0.001*** |
| PIR 1-3 |  | 3967 (35.62%) | 941 (32.60%) | 973 (34.59%) | 1002 (36.42%) | 1051 (38.86%) |  |
| PIR<1 |  | 1970 (14.25%) | 435 (12.73%) | 486 (14.04%) | 500 (14.75%) | 549 (15.47%) |  |
| PIR>3 |  | 3668 (50.13%) | 1025 (54.67%) | 935 (51.37%) | 893 (48.82%) | 815 (45.66%) |  |
| Smoke, n（%） | 9605 |  |  |  |  |  | <0.001*** |
| No |  | 7638 (80.31%) | 2051 (85.86%) | 1922 (81.52%) | 1875 (78.41%) | 1790 (75.44%) |  |
| Yes |  | 1967 (19.69%) | 350 (14.14%) | 472 (18.48%) | 520 (21.59%) | 625 (24.56%) |  |
| Drink, n（%） | 9605 |  |  |  |  |  | 0.05059 |
| No |  | 2252 (18.37%) | 583 (18.31%) | 595 (20.34%) | 553 (17.98%) | 521 (16.85%) |  |
| Yes |  | 7353 (81.63%) | 1818 (81.69%) | 1799 (79.66%) | 1842 (82.02%) | 1894 (83.15%) |  |
| Weight, n（%） | 9605 |  |  |  |  |  | <0.001*** |
| Normal |  | 2873 (30.37%) | 1142 (49.89%) | 824 (35.08%) | 561 (23.41%) | 346 (13.12%) |  |
| Overweight |  | 6560 (67.87%) | 1162 (46.08%) | 1528 (63.35%) | 1813 (75.79%) | 2057 (86.24%) |  |
| Underweight |  | 172 (1.76%) | 97 (4.02%) | 42 (1.57%) | 21 (0.8%) | 12 (0.64%) |  |
| Marital, n（%） | 9605 |  |  |  |  |  | <0.001*** |
| Married |  | 4900 (54.75%) | 1115 (52.44%) | 1190 (54.66%) | 1257 (53.78%) | 1338 (58.10%) |  |
| Never married |  | 1856 (19.25%) | 602 (22.62%) | 494 (20.22%) | 410 (18.48%) | 350 (15.69%) |  |
| Other |  | 2849 (26%) | 684 (24.94%) | 710 (25.12%) | 728 (27.74%) | 727 (26.21%) |  |
| Hypertension，n（%） | 9605 |  |  |  |  |  | <0.001*** |
| No |  | 5989 (66.07%) | 1685 (75.91%) | 1542 (68.45%) | 1421 (62.55%) | 1341 (57.38%) |  |
| Yes |  | 3616 (33.93%) | 716 (24.09%) | 852 (31.55%) | 974 (37.45%) | 1074 (42.62%) |  |
| Kidney Stone, n（%） | 9605 |  |  |  |  |  | <0.001*** |
| No |  | 8776 (91.07%) | 2237 (92.85%) | 2215 (92.68%) | 2167 (90.06%) | 2157 (88.7%) |  |
| Yes |  | 829 (8.93%) | 164 (7.15%) | 179 (7.32%) | 228 (9.94%) | 258 (11.3%) |  |
| Kidney Stone recurrence, n（%） | 6676 |  |  |  |  |  | <0.001*** |
| Yes |  | 180 (2.84%) | 31（1.97%） | 24（1.31%） | 48（3.22%） | 77（4.85%） |  |
| No |  | 6496 (97.16%) | 1629（98.03%） | 1646（98.69%） | 1607（96.78%） | 1614（95.15%） |  |

Quartile 1: TG/HDL-C<=1.17; Quartile 2: 1.17<TG/HDL-C<=1.88; Quartile 3: 1.88<TG/HDL-C<=3.13; Quartile 4: 3.13<TG/HDL-C

Continuous data are shown as mean (SE) and categorical data are shown as percentage.

*: 0.01<=P<0.05; **: 0.001<=P<0.01; ***: P<0.001

**Table S4**：Baseline characteristics according to HOMA-IR quartiles.

| Characteristic | Overall, N | HOMA-IR levels | | | | | |
| --- | --- | --- | --- | --- | --- | --- | --- |
|  |  | Overall | Q1 | Q2 | Q3 | Q4 | P |
| TyG index | 9605 | 8.54（0.00910） | 8.2 (0.0147) | 8.41 (0.0138) | 8.66 (0.0144) | 8.9 (0.0182) | <0.001*** |
| MET-IR | 9605 | 41.9（0.199） | 33.01 (0.215) | 37.98 (0.234) | 43.69 (0.259) | 52.81 (0.333) | <0.001*** |
| TG/HDL-C | 9605 | 2.68（0.0391） | 1.70 (0.0630) | 2.06 (0.0439) | 3.01 (0.0815) | 3.96 (0.106) | <0.001*** |
| HOMA-IR | 9605 | 2.96（0.0471） | 0.95 (0.00804) | 1.75 (0.00541) | 2.82 (0.0115) | 6.32 (0.0984) | <0.001*** |
| TyG-BMI index | 9605 | 244.50（1.06） | 196.91 (1.24) | 224.73 (1.28) | 254.6 (1.40) | 301.73 (1.75) | <0.001*** |
| SUA（mg/dl） | 9605 | 5.45（0.0199） | 5.01 (0.0402) | 5.22 (0.0316) | 5.51 (0.0369) | 6.04 (0.0373) | 0.0021** |
| VD3 | 9605 | 70.29（0.669） | 75.85 (1.03) | 72.92 (0.859) | 69.38 (0.880) | 63.08 (0.976) | 0.019* |
| Age, n（%） | 9605 |  |  |  |  |  | <0.001*** |
| 20-35 |  | 2908 (32.76%) | 749 (35.91%) | 752 (34.44%) | 721 (31.72%) | 686 (28.96%) |  |
| 35-65 |  | 4897 (52.88%) | 1107 (52.46%) | 1155 (50.52%) | 1237 (52.25%) | 1398 (56.28%) |  |
| >65 |  | 1800 (14.36%) | 358 (11.63%) | 450 (15.04%) | 514 (16.03%) | 478 (14.76%) |  |
| Gender, n (%) | 9605 |  |  |  |  |  | <0.001*** |
| Female |  | 4948 (51.18%) | 1167 (54.12%) | 1287 (55.25%) | 1257 (49.72%) | 1237 (45.63%) |  |
| male |  | 4657 (48.82%) | 1047 (45.88%) | 1070 (44.75%) | 1215 (50.28%) | 1325 (54.37%) |  |
| Race, n（%） | 9605 |  |  |  |  |  | <0.001*** |
| Mexican American |  | 1386 (7.96%) | 226 (4.58%) | 304 (6.44%) | 414 (9.47%) | 442 (11.35%) |  |
| Non-Hispanic Black |  | 1736 (9.92%) | 678 (9.30%) | 504 (10.12%) | 354 (9.78%) | 200 (10.50%) |  |
| Non-Hispanic White |  | 4412 (69.56%) | 999 (73.91%) | 1095 (70.58%) | 1107 (67.23%) | 1211 (66.54%) |  |
| Other Hispanic |  | 959 (5.26%) | 191 (4.34%) | 233 (5.82%) | 244 (5.04%) | 291 (5.85%) |  |
| Other Race |  | 1112 (7.29%) | 307 (7.87%) | 258 (7.04%) | 276 (8.49%) | 271 (5.76%) |  |
| Education, n（%） | 9605 |  |  |  |  |  | 0.1416 |
| Below high school |  | 1129 (8.48%) | 250 (8.02%) | 267 (8.06%) | 304 (8.56%) | 308 (9.29%) |  |
| College or above |  | 7129 (76.77%) | 1660 (79.28%) | 1757 (76.41%) | 1817 (76.13%) | 1895 (75.27%) |  |
| High school |  | 1347 (14.75%) | 304 (12.71%) | 333 (15.53%) | 351 (15.32%) | 359 (15.44%) |  |
| PIR, n（%） | 9605 |  |  |  |  |  | <0.001*** |
| PIR 1-3 |  | 3967 (35.62%) | 869 (33.4%) | 966 (34.15%) | 1039 (36.42%) | 1093 (38.51%) |  |
| PIR<1 |  | 1970 (14.25%) | 412 (12.99%) | 440 (13.01%) | 524 (14.53%) | 594 (16.46%) |  |
| PIR>3 |  | 3668 (50.13%) | 933 (53.62%) | 951 (52.83%) | 909 (49.05%) | 875 (45.03%) |  |
| Smoke, n（%） | 9605 |  |  |  |  |  | 0.00196** |
| No |  | 7638 (80.31%) | 1680 (77.22%) | 1865 (79.7%) | 2018 (82.18%) | 2075 (82.12%) |  |
| Yes |  | 1967 (19.69%) | 534 (22.78%) | 492 (20.3%) | 454 (17.82%) | 487 (17.88%) |  |
| Drink, n（%） | 9605 |  |  |  |  |  | <0.001*** |
| No |  | 2252 (18.37%) | 455 (15.46%) | 555 (18.07%) | 575 (18.92%) | 667 (21.03%) |  |
| Yes |  | 7353 (81.63%) | 1759 (84.54%) | 1802 (81.93%) | 1897 (81.08%) | 1895 (78.97%) |  |
| Weight, n（%） | 9605 |  |  |  |  |  | <0.001*** |
| Normal |  | 2873 (30.37%) | 1307 (58.51%) | 897 (37.69%) | 501 (18.74%) | 168 (6.56%) |  |
| Overweight |  | 6560 (67.87%) | 783 (36.42%) | 1424 (60.85%) | 1962 (80.84%) | 2391 (93.35%) |  |
| Underweight |  | 172 (1.76%) | 124 (5.07%) | 36 (1.46%) | 9 (0.42%) | 3 (0.09%) |  |
| Marital, n（%） | 9605 |  |  |  |  |  | 0.07622 |
| Married |  | 4900 (54.75%) | 1061 (53.74%) | 1178 (52.51%) | 1303 (57.15%) | 1358 (55.57%) |  |
| Never married |  | 1856 (19.25%) | 499 (20.93%) | 470 (19.70%) | 453 (18.76%) | 434 (17.62%) |  |
| Other |  | 2849 (26%) | 654 (25.33%) | 709 (27.79%) | 716 (24.08%) | 770 (26.81%) |  |
| Hypertension, n（%） | 9605 |  |  |  |  |  | <0.001*** |
| No |  | 5989 (66.07%) | 1619 (77.97%) | 1586 (71.04%) | 1473 (62.27%) | 1311 (53.01%) |  |
| Yes |  | 3616 (33.93%) | 595 (22.03%) | 771 (28.96%) | 999 (37.73%) | 1251 (46.99%) |  |
| Kidney Stones, n（%） | 9605 |  |  |  |  |  | <0.001*** |
| No |  | 8776 (91.07%) | 2077 (93.72%) | 2176 (92.31%) | 2225 (89.31%) | 2298 (88.94%) |  |
| Yes |  | 829 (8.93%) | 137 (6.28%) | 181 (7.69%) | 247 (10.69%) | 264 (11.06%) |  |
| Kidney Stone recurrence, n（%） | 6676 |  |  |  |  |  | 0.002315** |
| Yes |  | 180 (2.84%) | 21（1.46%） | 34（1.73%） | 54（3.8%） | 71（4.33%） |  |
| No |  | 6496 (97.16%) | 1498（98.54%） | 1604（98.27%） | 1652（96.2%） | 1742（95.67%） |  |

Quartile 1: HOMA-IR<=1.37; Quartile 2: 1.37<HOMA-IR<=2.19; Quartile 3: 2.19<HOMA-IR<=3.63; Quartile 4: 3.63<HOMA-IR

Continuous data are shown as mean (SE) and categorical data are shown as percentage.

*: 0.01<=P<0.05; **: 0.001<=P<0.01; ***: P<0.001

**Table S5**：Baseline characteristics according to TyG-BMI quartiles.

| Characteristic | Overall, N | TyG-BMI index | | | | | |
| --- | --- | --- | --- | --- | --- | --- | --- |
|  |  | Overall | Q1 | Q2 | Q3 | Q4 | P |
| TyG index | 9605 | 8.54（0.00910） | 8.08 (0.0120) | 8.43 (0.0136) | 8.72 (0.0122) | 8.94 (0.0170) | <0.001*** |
| MET-IR | 9605 | 41.9（0.199） | 29.4 (0.101) | 36.8 (0.0840) | 43.8 (0.0931) | 57.6 (0.304) | <0.001*** |
| TG/HDL-C | 9605 | 2.68（0.0391） | 1.28 (0.0240) | 2.01 (0.0393) | 3.02 (0.0552) | 4.41 (0.124) | <0.001*** |
| HOMA-IR | 9605 | 2.96（0.0471） | 1.47 (0.0313) | 2.09 (0.0399) | 3.08 (0.0544) | 5.20 (0.116) | <0.001*** |
| TyG-BMI index | 9605 | 244.50（1.06） | 175.06 (0.0475) | 217.44 (0.275) | 255.59 (0.0326) | 329.87 (1.57) | <0.001*** |
| SUA（mg/dl） | 9605 | 5.45（0.0199） | 4.75 (0.0324) | 5.27 (0.0349) | 5.70 (0.0312) | 6.06 (0.0334) | 0.0032** |
| VD3 | 9605 | 65.76（27.21） | 74.31 (1.03) | 73.96 (0.796) | 69.49 (0.969) | 63.44 (0.971) | <0.001*** |
| Age, n（%） | 9605 |  |  |  |  |  | <0.001*** |
| 20-35 |  | 2908 (32.76%) | 1003（45.55%） | 649（29.84%） | 602（25.89%） | 654（29.75%） |  |
| 35-65 |  | 4897 (52.88%) | 950（43.08%） | 1253（52.8%） | 1327（57.32%） | 1367（58.88%） |  |
| >65 |  | 1800 (14.36%) | 360（11.37%） | 541（17.37%） | 539（16.79%） | 360（11.93%） |  |
| Gender, n (%) | 9605 |  |  |  |  |  | <0.001*** |
| Female |  | 4948 (51.18%) | 1358（60.79%） | 1150（48.12%） | 1126（43.69%） | 1314（52.12%） |  |
| male |  | 4657 (48.82%) | 955（39.21%） | 1293（51.88%） | 1342（56.31%） | 1067（47.88%） |  |
| Race, n（%） | 9605 |  |  |  |  |  | <0.001*** |
| Mexican American |  | 1386 (7.96%) | 202（5.04%） | 317（6.94%） | 450（10.1%） | 417（9.75%） |  |
| Non-Hispanic Black |  | 1736 (9.92%) | 408（9.89%） | 432（9.49%） | 429（9.38%） | 467（10.93%） |  |
| Non-Hispanic White |  | 4412 (69.56%) | 1080（69.78%） | 1118（70.02%） | 1108（69.73%） | 1106（68.73%） |  |
| Other Hispanic |  | 959 (5.26%) | 177（4.57%） | 259（5.2%） | 278（5.99%） | 245（5.3%） |  |
| Other Race |  | 1112 (7.29%) | 446（10.72%） | 317（8.35%） | 203（4.8%） | 146（5.3%） |  |
| Education, n（%） | 9605 |  |  |  |  |  | <0.001*** |
| Below high school |  | 1129 (8.48%) | 231（7.64%） | 287（7.92%） | 316（9.21%） | 295（9.16%） |  |
| College or above |  | 7129 (76.77%) | 1786（79.75%） | 1834（77.91%） | 1792（75.34%） | 1717（74.09%） |  |
| High school |  | 1347 (14.75%) | 296（12.61%） | 322（14.17%） | 360（15.45%） | 369（16.75%） |  |
| PIR, n（%） | 9605 |  |  |  |  |  | <0.001*** |
| PIR 1-3 |  | 3967 (35.62%) | 883（33%） | 988（34.11%） | 1073（36.86%） | 1023（38.5%） |  |
| PIR<1 |  | 1970 (14.25%) | 481(15.13%) | 449（12.43%） | 477（13.34%） | 563（16.09%） |  |
| PIR>3 |  | 3668 (50.13%) | 949(51.87%) | 563（53.46%） | 918（49.79%） | 795（45.41%） |  |
| Smoke, n（%） | 9605 |  |  |  |  |  | 0.01695* |
| No |  | 7638 (80.31%) | 1797（78.25%） | 1945（79.04%） | 1992(81.29%) | 1904(82.64%) |  |
| Yes |  | 1967 (19.69%) | 516（21.75%） | 498（20.96%) | 476(18.71%) | 477（17.36%） |  |
| Drink, n（%） | 9605 |  |  |  |  |  | 0.1718 |
| No |  | 2252 (18.37%) | 562（18.82%） | 552（17.45%） | 542（17.35%) | 596（19.85%） |  |
| Yes |  | 7353 (81.63%) | 1751（81.18%） | 1891（82.55%） | 1926（82.65%） | 1785（80.15%） |  |
| Weight, n（%） | 9605 |  |  |  |  |  | <0.001*** |
| Normal |  | 2873 (30.37%) | 2046（89.19%） | 801（31.74%） | 26（0.56%） | 0 |  |
| Overweight |  | 6560 (67.87%) | 95（3.78%） | 1642（68.26%） | 2442（99.44%） | 2381 |  |
| Underweight |  | 172 (1.76%) | 172（7.03%） | 0 | 0 | 0 |  |
| Marital, n（%） | 9605 |  |  |  |  |  | <0.001*** |
| Married |  | 4900 (54.75%) | 1047（49.41%） | 1264（54.83%） | 1358（58.18%） | 1231（56.56%） |  |
| Never married |  | 1856 (19.25%) | 681（27.88%） | 414（17.39%） | 345（14.56%） | 416（17.19%） |  |
| Other |  | 2849 (26%) | 585（22.71%） | 765（27.79%） | 765（27.26%） | 734（26.25%） |  |
| Hypertension, n（%） | 9605 |  |  |  |  |  | <0.001*** |
| No |  | 5989 (66.07%) | 1803（82.09%） | 1587（68.78%） | 1406（61.22%） | 1193（52.2%） |  |
| Yes |  | 3616 (33.93%) | 510（17.91%） | 856（31.22%） | 1062（38.78%） | 1188（47.8%） |  |
| Kidney Stones, n（%） | 9605 |  |  |  |  |  | <0.001*** |
| No |  | 8776 (91.07%) | 2184（94.43%） | 2259（92.18%） | 2214（88.85%） | 2119（88.82%） |  |
| Yes |  | 829 (8.93%) | 129（5.59%） | 184（7.82%） | 254（11.15%） | 262（11.18%） |  |
| Kidney Stone recurrence, n（%） | 6676 |  |  |  |  |  | <0.001*** |
| Yes |  | 180 (2.84%) | 21（1.18%） | 39（2.39%） | 60（3.75%） | 60（4.18%） |  |
| No |  | 6496 (97.16%) | 1630(98.82%) | 1704（97.61%） | 1638(96.25%) | 1524（95.82%） |  |

Quartile 1: TyG-BMI<=198.62; Quartile 2: 198.62<TyG-BMI<=234.98; Quartile 3: 234.98<TyG-BMI<=278.67; Quartile 4: 278.67<TyG-BMI

Continuous data are shown as mean (SE) and categorical data are shown as percentage.

*: 0.01<=P<0.05; **: 0.001<=P<0.01; ***: P<0.001
